# Fungi and Aflatoxin Levels in Traditionally Processed Cassava (*Manihot esculenta* Crantz) Products in Homa Bay County, Kenya

**DOI:** 10.1155/2020/3406461

**Published:** 2020-08-26

**Authors:** Boniface Oure Obong'o, George Ayodo, Fanuel Kawaka, Morelly Kathy Adalla

**Affiliations:** ^1^School of Health Sciences, Jaramogi Oginga Odinga University of Science and Technology, P.O. Box 210, Bondo, Kenya; ^2^Kenya Industrial Research and Development Institute, P.O. Box 6017, Kisumu, Kenya; ^3^School of Applied and Health Sciences, Technical University of Mombasa, P.O. Box 90420, Mombasa, Kenya

## Abstract

Cassava (*Manihot esculenta* Crantz) is a major source of carbohydrates, calcium, vitamins (B and C), and essential minerals and is the third most important source of calories in the tropics. However, it is not clear if the traditional processing methods expose the products to microbial contamination. This study assessed the levels of fungi and aflatoxin contamination in traditionally processed cassava products (*Akuoga* and *Abeta*). A total of 38 samples were collected from the local markets in 7 subcounties in Homa Bay County, Kenya. The levels of aflatoxin were determined using an indirect competitive ELISA protocol. Yeast and mould contamination was determined using ISO 21527-2 method. Mean aflatoxin levels in chopped, fermented, and sun-dried cassava (*Akuoga)* were 0.36 *μ*g/kg compared to 0.25 *μ*g/kg in chopped and sun-dried (*Abeta)* products. Aflatoxin contamination was detected in 55% of the samples and ranged from 0–5.33 *μ*g/kg. These levels are within 10 *μ*g/kg recommended by the CODEX STAN 193-1995. Yeast and mould counts in fermented and chopped sun-dried products were 3.16 log Cfu/g and 2.92 log Cfu/g, respectively. The yeast and mould counts were above standards set by East African Standard 739:2010 in 62% (*Akuoga*) and 58% (*Abeta*). The most prevalent fungal species were *Saccharomyces cerevisiae* (68.4%) and *Candida rugosa* (68%) followed by *Candida parapsilosis* (18.4%), *Candida tropicalis* (15.8%)*, Candida humilis* (15.8%), and *Aspergillus* spp. (5.3%). *Aspergillus* spp. was the only mycotoxigenic mould isolated from the samples. The study shows that cassava consumers are exposed to the risk of aflatoxin poisoning. The study, therefore, recommends appropriate surveillance to ensure safety standards.

## 1. Introduction

Cassava (*Manihot esculenta* Crantz) is produced in more than 100 countries and fulfils the daily caloric demands of millions of people living in tropical America, Africa, and Asia. Its importance as a food security crop is dominant in Western, Central, and Eastern Africa due to its ability to produce a good yield (∼10 t/ha) in marginal areas with poor soils and with minimal inputs [1]. In Kenya, cassava is mainly grown in the western, coastal, eastern, and central regions [2]. The western region grows and consumes 60% of the national cassava production, which stood at 946,076 tonnes in 2018 [3]. The cassava crop is mainly produced by small-scale farmers using traditional farming methods [4]; consumption and utilisation of the crop increases during drought or when other major staple stocks are depleted. The crop is viewed as a reserve commodity. However, of late, there has been increased processing of cassava into flour for commercial sale in supermarkets and open-air markets [5]. In Homa Bay County just like other parts of western Kenya, cassava is processed into sun-dried cassava chips locally referred to as *Abeta* and fermented cassava crumps referred to as *Akuoga*. The processing is mostly done at the household level using simple tools and equipment. The traditional processing of cassava into various indigenous products plays a vital role in the food supply chain by transforming the crop into a stable product with reduced toxicity and improved palatability and hence reduced postharvest losses [6].

*Akuoga* is prepared through solid-state fermentation. The process involves peeling, washing, slicing, and surface drying then placing in sacks or large baskets and storing in a dark, cold room for 3 to 4 days. Weights are added on top of the sacks or baskets to facilitate draining of water from the fermenting cassava. During fermentation, the cassava develops moulds (black/grey colouration) and become soft. The cassava which now resembles pulp is removed from the sack and placed on mats to sun-dry. The development of green coloured mould would indicate insufficient first surface drying step [7]. *Abeta*, on the other hand, is prepared by peeling, washing, slicing, and drying, and the processed and dried cassava chips are then milled into flour [2].

Cassava is not generally associated with mould and aflatoxin contamination when fresh, due to the high moisture content and the presence of antiaflatoxin compounds [8]. Hence, it can be considered as a crop that is resistant to aflatoxin contamination during the preharvest period [9]. However, processing, for example, sun-drying, reduces the moisture content, hence exposing the crop to contamination by fungi following moisture reabsorption. During fermentation *Aspergillus* spp. may produce aflatoxins under the right conditions of humidity, temperature, and pH [8, 10]. A study by Gacheru et al. found out that 87.5% of dried cassava in Nairobi, Kenya, had yeast and mould count bellow 3.0 Cfu g^−1^, while in the coastal region, 60% of the samples had yeast and mould counts above the set limit [7]. They attributed the results to the high humidity levels in the coastal region. Yeast, mould, and aflatoxin contamination of cassava is much dependent on processing practices, storage facilities, and the duration of storage [11].

The cassava processing methods reduce the cyanide content, improve palatability, and increase the shelf life of the highly perishable roots [12]. However, drying of cassava is often done on the ground, and the products are exposed to contamination with soil, dust, moulds, and other foreign matter. The practice promotes contact between the products and the soil, which is a primary source of moulds [8]. Despite the observations, limited studies have focused on the effect of traditional cassava processing methods on fungal and aflatoxin levels in western Kenya where cassava is dominantly produced and consumed. This study established the fungal load and aflatoxin contamination levels of the traditionally processed cassava products available in the local markets.

## 2. Materials and Methods

### 2.1. Study Site

**Figure 1:**
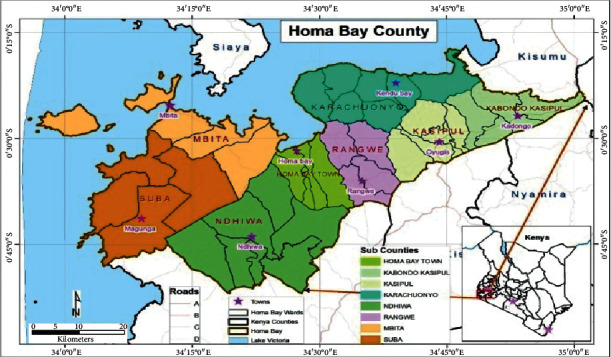
Subcounties in Homa Bay County (source: GoK [13]).

The study was carried out in 7 subcounties in Homa Bay County within the western Kenya region (Figure 1). The county was selected based on agroclimatic conditions and prevalence of cassava cultivation and consumption.

### 2.2. Sample Collection

Cassava samples were collected from April 2018–May 2018 from vendors in the main local markets in the seven subcounties using the FAO aflatoxin Sampling Protocol previously described by Fonseca [14]; sample size determination was done as per equation (1). Approximately 500 g of both *Akuoga* and *Abeta* was purchased from all vendors in the sampling locations based on availability, thereafter combined to form the bulk samples. The samples were then packed in airtight polythene carrier bags to prevent contamination and moisture uptake and transported to the Kenya Industrial Research and Development Institute (KIRDI) Laboratories in Kisumu for analysis. The bulk samples were mixed to attain homogeneity and then milled and subdivided into three subsamples of equal weight (100 g) using a rotary sample divider. The resultant working samples were then analyzed individually.

(1) $$\begin{array}{c} \text{NS}=4\sqrt{\text{SL}}\;, \end{array}$$

where

  NS = minimum number of sacks to be sampled
  SL = number of sacks of a lot
  The sampling locations (7 subcounties) were considered as lots having an avereage of 15 sacks each for Abeta and Akuoga procducts
  NS = 4√15
  NS = 15.4
  Sample size: 2 bulked samples per location (2 00D7 7) = 14
  One location only yielded one bulk sample = 13
  13 × 3 working samples for every bulked sample = 13 × 3 = 39
  One sample was eliminated due to contamination = 38
  Final sample size: 38

Samples of each cassava products were collected from all the traders in the major markets in the seven subcounties and combined to form one bulk sample for every product type per sampling location resulting in thirteen bulk samples because one location only had one product type. The bulk samples were homogenized and divided into three subsamples each using a rotary sample divider. The resulting 39 working samples were analyzed individually. One sample was grossly cross contaminated during analysis and discarded leaving a total of 38 samples.

### 2.3. Sample Preparation and Culturing

Approximately 15 g of each of the sampled cassava products was ground using a laboratory sample mill (Ramtons, model: RM/519, China) into fine flour. The ground flour was used for the isolation, identification, and enumeration of the yeast and mould. For each sample, 0.1 ml of 10^−1^ to 10^−5^ dilutions was aseptically spread plated using a 90° sterile glass spreader on 90 mm Petri dishes containing Dichloran 18% (mass concentration) glycerol agar (DG18) supplemented with chloramphenicol prepared in duplicates. The plates were incubated in an upright position with lids uppermost in an incubator at 25°C ± 1°C for 5 to 7 days. After incubation, colonies that grew on the plates were counted with the aid of a colony counter. Colonies that appeared flat, fluffy with coloured or sporulating structures were enumerated as moulds and expressed as colony forming units (Cfu) per gram of the cassava sample using the general equation below.

(2) $$\begin{array}{c} N=\frac{{\sum c}^{}}{V\times 1.1\times d}, \end{array}$$

where ∑*c* is the sum of the colonies counted on two dishes retained from two successive dilutions, at least one of which contains a minimum of 10 colonies to more than 150 colonies; *V* is the volume of inoculum placed in each dish, in millilitres; and *d* is the dilution corresponding to the first dilution retained.

### 2.4. Isolation and Identification of Yeasts and Moulds

Following incubation, the resulting growth was examined for the presence of discrete colonies which were then purified by repeated subculturing three times on fresh sterile DG18 agar plates and incubated as earlier described. The resulting culture plates were examined for uniformity of growth as an indication of purity. DG18 bottle slants were subsequently prepared and stored in a refrigerator (4°C) for characterization and identification. A collection of 20 isolates was constituted and observed microscopically.

Yeasts were identified by examining key features of yeast colonies on DG18 and cell morphology after lactophenol cotton blue staining observed under X40 objective lens on a compound microscope. Biochemical characterization was carried out using standard taxonomical methods [15, 16]. The biochemical tests carried out include acid production from glucose, urea hydrolysis, tolerance to 1% acetic acid, and fermentation of different sugars. The yeast isolates were identified by comparing with already described yeasts in Pitt and Hocking [16]. Mould identification was made through macroscopic and microscopic observations of the cultures. The mycelia's physical characteristics such as colour, structure, and shape were noted as well as the microscopic characteristics as previously described by Pitt and Hocking [16]. Some morphological structures used for identification included septation, presence/absence of sporangiophores, fruiting bodies, and other special organs like the rhizoids. Yeast and moulds that could not be identified using the available keys were categorised as others.

### 2.5. Aflatoxin Analysis

Representative samples were ground to the particle size of fine instant coffee using an analytical laboratory grinder (Retsch, Model: DM 200, Haan, Germany) and mixed thoroughly. Two grams of the homogenized sample was weighed into a suitable container, and then 10 ml of 70% methanol was added. The solution was mixed at room temperature for 10 minutes by shaking manually and then centrifuged for 10 minutes at 3500 g at room temperature using a centrifuge (Beckman, model: Allegra 64R, Palo Alto, USA). Microtiter wells for standards and samples were placed into microwell holders, and the positions were recorded. In each well, 50 *μ*l of the standards and samples was added to separate duplicate wells. About 50 *μ*l of the conjugate was added to all the wells followed by 50 *μ*l of antibody to each well, shaken gently and incubated for 30 min at room temperature (20–25°C). The liquid was poured, and the plate was tapped upside down against an absorbent paper vigorously to ensure complete removal of the liquid from the wells. The wells were then filled with 250 *μ*l of wash buffer and then emptied. The washing was repeated twice, and 100 *μ*l of the substrate was added to each well and mixed gently by shaking after which the plates were incubated at 20–25°C for 15 minutes.

After incubation, 100 *μ*l of stop solution was added to each well and mixed gently by shaking the plate manually. The optical density of each microwell was read with an ELISA microtiter plate reader at 450 nm wavelength. The % absorbance was then calculated as follows:

(3) $$\begin{array}{c} \%\text{absorbance}=\frac{\text{absorbance of standard or sample}}{\text{absorbance of zero standard}}\times 100. \end{array}$$

The values calculated for the standards were entered in a system of coordinates on semilogarithmic graph paper against the aflatoxin concentration (*μ*g/kg). In order to obtain the actual aflatoxin concentration in *μ*g/kg contained in the sample, the concentration read from the calibration curve was multiplied by the corresponding dilution factor 35 (dilution factor for cereals and feed) when working in accordance with the regulations stated. The samples were tested using the Ridascreen aflatoxin total enzyme-linked immunosorbent assay (ELISA) kit. The limit of detection was 1.75 *μ*g/kg. The samples were run in duplicates [17].

### 2.6. Statistical Analysis

The total quantity of fungi from the samples was calculated as colony forming unit per gram (Cfu/g) and described as a percentage using morphological characteristics [16]. Quantitative data (aflatoxin levels and yeast and mould counts) were subjected to ANOVA using SPSS statistical programme version 24. The mean was separated using LSD (*p* < 0.05). *p* value < 0.05 was considered statistically significant.

### 2.7. Ethical Consideration

Ethical clearance was sought from the Jaramogi Oginga Odinga Teaching and Referral Hospital Ethical Review Committee (ERC.1B/VOL.1/400).

## 3. Results

### 3.1. Yeast and Mould Contamination

**Figure 2:**
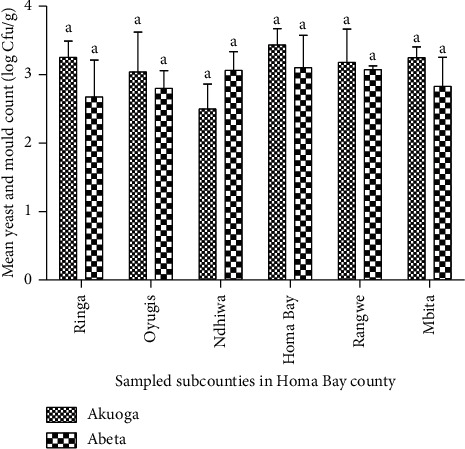
Yeast and mould count in A*kuoga* and *Abeta* types of processed cassava. Figures with same letter are not significantly different.

The analysis of yeast and mould counts in the samples indicated that the mean counts in Abeta products was 2.92 log Cfu/g and 3.16 log Cfu/g in Akuoga products (Table 1). Among the subcounties, higher mean mould counts were recorded in Suba (3.47 log Cfu/g) and Homa Bay (3.27 log Cfu/g) compared to Ndhiwa at 2.78 log Cfu/g (Table 2). In Homa Bay and Ringa subcounties, Akuoga products recorded higher mould counts of 3.47 log Cfu/g and 3.44 log Cfu/g, respectively (Figure 2). Ndhiwa subcounty had the lowest mean mould counts in Akuoga at 2.50 log Cfu/g, followed by *Abeta* (2.68 log Cfu/g) in Ringa and Oyugis (2.80 log Cfu/g).

### 3.2. Diversity of Yeast and Mould in Products

Characterization of the isolated yeast and moulds showed the presence of different species in the cassava products (Table 3). The results showed that in Akuoga, *Candida rugosa* was the most prevalent at 44.7% followed by *Saccharomyces cerevisiae* (39.5), *Candida parapsilosis* (15.8%), *Candida tropicalis* (15.8%)*, Candida humilis* (7.9%), and *Aspergillus* spp. (5.3%). In Abeta, the higher prevalence was observed in *Saccharomyces cerevisiae* (28.9%) followed by others (26.3%), *Candida rugosa* (23.7%), *Candida* humilis (7.9%), *Candida albicans* (5.3%), and *Candida parapsilosis* (2.6%). No *Candida tropicalis* and *Aspergillus* spp. were isolated in Abeta with 26.3% of the organisms remaining unidentified and were thus categorised as others.

### 3.3. Aflatoxin Levels in the Products

**Figure 3:**
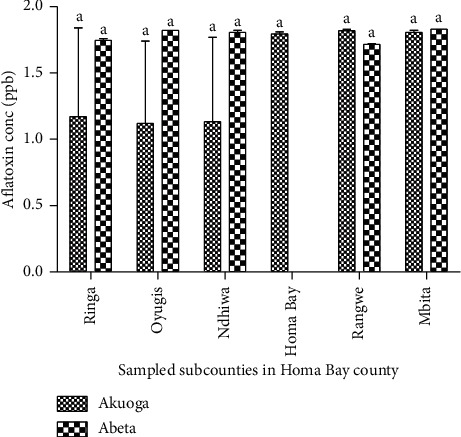
Aflatoxin quantification in *Akuoga* and *Abeta* types of processed cassava. Figures with same letter are not significantly different.

Aflatoxin levels were high in Akuoga (0.36 *μ*g/kg) compared to 0.25 0.36 *μ*g/kg) in Abeta (Table 4). In the subcounties, high levels of aflatoxin were recorded in Mbita (0.35 *μ*g/kg) compared to the other counties at 0.25 *μ*g/kg (Table 5). Among the Akuoga products, Mbita subcounty recorded a higher level of aflatoxin at 0.35 *μ*g/kg compared to 0.24 *μ*g/kg in Kasipul (Figure 3). The grading of cassava products according to the extent of visible mouldiness comparative to the levels of aflatoxin contamination showed that nonmouldy products recorded high levels of aflatoxin compared to slight and moderate moulding products (Table 6).

## 4. Discussion

### 4.1. Yeast and Mould Contamination

This study shows that yeast and mould contamination in fermented cassava products exceeded the set limits of 3.0 log Cfu/g by the East African Standards EAS 739:2010. The high levels of mould contamination observed in the *Akuoga* compared to *Abeta* could be due to prolonged exposure to soil particles during the process of drying. This practice promotes contact between the cassava products and the soil, which is a primary source of moulds, dust, and other foreign matters [18]. During drying, Akuoga products are usually spread on soil for longer periods to completely dry compared to Abeta. Similar studies have reported that the process of fermentation favours the growth of several organisms including *Aspergillus fumigatus, A. niger*, and *A. flavus*, in particular, high humidity, temperature, and favourable pH of 5-6 conditions [19–21]. A survey by Kaaya and Eboku [22] demonstrated that in Kumi district, Eastern Uganda, fermented cassava chips are more contaminated with moulds and yeasts than nonfermented. In another study, Gacheru et al. [7] showed that cassava samples from the coastal regions had higher yeast and moulds than mainland Nairobi, Kenya. These authors attributed their findings to the elevated levels of humidity often associated with large water bodies. Our study focused on subcounties within four agroecological zones (UM1, LM2, LM3, LM4, and LM5) along Lake Victoria basin with elevated levels of varying humidity, which is a possible contribution to the unclear trends of occurrence of moulds and yeasts in the cassava products.

### 4.2. Diversity of Yeast and Mould in Products

The high prevalence of yeast and moulds observed in fermented cassava products (*Akuoga*) has been reported in many studies [23]. The isolation of diverse microorganisms from fermented products could be due to the use of mixed starter cultures from previous batches. The presence of different groups of organisms in fermented cassava products could also be an indication of possible coexistence. Growth of yeasts in fermented foods is favoured by the acidification of the environment by bacteria, and in turn, yeasts provide growth factors such as vitamins and soluble nitrogen compounds for the bacteria [19, 24]. The occurrence of diverse groups of yeasts in the products could, therefore, be associated with the breaking down of starch to increase the nutritional value of fermented and nonfermented cassava. The high prevalence of *Candida* and *Saccharomyces* in the products is consistent with studies elsewhere that reported them as the most dominant yeasts in many foods in sub-Saharan Africa [25, 26]. Reports have shown that *S. cerevisiae* is the dominant yeast in the fermentation of most indigenous food products in sub-Saharan Africa [27, 28]. The isolation of *Aspergillus* spp. from both fermented and unfermented cassava products has been widely reported by many authors [8, 12, 20, 29, 30] and has been classified as one of the principal genera that contaminate cassava together with *Fusarium* and *Alternaria* [8, 31]. The dominant organisms in the fermented products could be exploited to replace the use of traditional starter culture and improve the quality of the final product.

### 4.3. Aflatoxin Levels in the Products

The cassava products had aflatoxin levels that were below the 10 *μ*g/kg regulatory limit for aflatoxin levels set by the Kenya Bureau of Standards. Low levels are probably due to good drying environments after processing that allows rapid drying of the products, thus limiting fungal growth and possible contamination. Varying levels of aflatoxin was detected in all fermented and nonfermented products. Cassava products sold in local markets are usually transported from smallholder farmers, and contamination could easily occur during transportation. The markets are mostly open-air, with no proper storage facilities. Transporting food products with inappropriate storage facilities from one location to another is known to favour aflatoxin contamination [32]. Several studies in other countries have also reported aflatoxin contamination of other food products at the market level [33, 34]. In a previous study, storage has been described as a factor that would lead to increases in aflatoxin after harvest [35]. On visual grading, mouldy cassava products showed reduced levels of aflatoxin compared to the nonmouldy products. The lactic acid bacteria (LAC) involved in natural fermentation has been reported to remove aflatoxin from most raw products effectively [36, 37]. LAC removes toxins through noncovalent binding of mutagens by fractions of its cell wall [38]. Live microorganisms absorb aflatoxin by attaching it to their cell walls or through active internalisation and accumulation [39]. Moreover, some studies have demonstrated the ability of bacteria like *Bacillus subtilis* to reduce the production of aflatoxin B and G by *Aspergillus parasiticus* during fermentation [40]. The detection of aflatoxin in nonmouldy cassava products could be attributed to microbial transition during fermentation where the acidic pH favours yeast than moulds.

## 5. Conclusions

The study has demonstrated that fermented and nonfermented cassava products sold in the open-air markets of western Kenya harbour a large diversity of moulds and yeasts. These microorganisms included *Saccharomyces cerevisiae, Candida rugosa, Candida parapsilosis, Candida tropicalis, Candida humilis*, and *Aspergillus* spp. There is a need for strict continuous monitoring and regulatory standards to ensure that aflatoxin contamination in cassava products does not exceed the 10 *μ*g/kg regulatory limit set by the Kenya Bureau of Standards (KEBS). Stakeholder awareness creation at all levels along the cassava value chain should be emphasised to ensure safe handling and reduction in contamination especially for smallholder farmers, transporters, traders, and even consumers. The dominant microorganisms isolated from the fermented products should be exploited to improve traditional starter culture for quality cassava products. Visual grading also demonstrated that mouldy products had reduced levels of aflatoxin contamination and should, therefore, be considered as surveillance.

## Figures and Tables

**Table 1:** Yeast and mould counts in *Abeta* and *Akuoga* cassava products.

Cassava product	Mean ± std error (log Cfu/g)	95% CI
*Akuoga*	3.16 ± 0.13	2.89–3.43
*Abeta*	2.92 ± 0.14	2.64–3.21

**Table 2:** Mean yeast and mould count of cassava products in different subcounties.

Subcounty-market	Mean ± std error (log Cfu/g)	95% CI
Kabondo-Ringa	2.97 ± 0.29	2.37–3.56
Kasipul-Oyugis	2.93 ± 0.29	2.34–3.51
Ndhiwa-Ndhiwa	2.78 ± 0.24	2.30–3.27
Homa Bay-Municipal	3.27 ± 0.25	2.77–3.77
Rangwe-Rangwe	3.15 ± 0.27	2.61–3.69
Mbita-Mbita	3.04 ± 0.22	2.58–3.49
Suba-Nyandiwa	3.47 ± 0.33	2.79–4.15
LSD *p* > 0.05	NS	

NS = means were not significantly different.

**Table 3:** Incidence of yeast and mould in the cassava products.

Yeast and mould species	Cassava products	
	*Akuoga*	*Abeta*
	Incidence (%)	Incidence (%)
*Candida rugosa*	44.7	23.7
*Candida humilis*	7.9	7.9
*Candida tropicalis*	15.8	0
*Candida albicans*	0	5.3
*Saccharomyces cerevisiae*	39.5	28.9
*Candida parapsilosis*	15.8	2.6
*Aspergillus* spp.	5.3	0
Others	0	26.3

**Table 4:** Aflatoxin content of *Abeta* and *Akuoga*.

Cassava product	Mean ± std error (*μ*g/kg)	(95% CI)
*Akuoga*	0.36 ± 0.07	0.22–0.50
*Abeta*	0.25 ± 0.00	0.25–0.26

**Table 5:** Mean aflatoxin content in the subcounties.

Subcounty-market	Mean ± std error (*μ*g/kg)	(95% CI)
Homa Bay-Municipal	0.25 ± 0.004	0.25–0.26
Mbita-Mbita	0.35 ± 0.093	0.16–0.55
Ndhiwa-Ndhiwa	0.25 ± 0.004	0.25–0.26
Kasipul-Oyugis	0.25 ± 0.007	0.24–0.27
Rangwe-Rangwe	0.25 ± 0.007	0.23–0.26
Kabondo-Ringa	0.25 ± 0.008	0.23–0.27

**Table 6:** Relationship between aflatoxin levels and degree of moulding.

Visual grading	% positive samples	Mean aflatoxin (*μ*g/kg)
Non	81.6	0.31
Slight	13.2	0.25
Moderate	5.3	0.26
Heavy	0	0
LSD (*p* > 0.05)		NS

NS = means were not significantly different.

## Data Availability

All the necessary data required for replication of this work and/or conducting secondary analysis are included within the article.
